# Logics and Agency in Public Management Research

**DOI:** 10.1007/s11115-021-00576-8

**Published:** 2022-09-27

**Authors:** Tony Kinder, Jari Stenvall, Antti Talonen

**Affiliations:** 1 Administrative Science, Tampere University, Tampere, Finland; 2 Faculty of Law, University of Helsinki, Helsinki, Finland

**Keywords:** Logics, Agency, Public organization, Public management

## Abstract

The article analyses the negative effects of the use of logics in public organisation research on active human agency. We build up a new conceptual model with which to approach logics in current research on organising public services; suggesting ways in which current models using logics in public organisation research can be strengthened. Our contribution is two-fold: we argue that Elder-Vass’ approach benefits from close synthesis with social learning theory (including recent thinking on trust, emotions, and distributed learning) and secondly, that grounding all usage of logics in logic-of-practice helps avoid a reification of logics and thirdly that situated learning better suits public organisation problem solving that the application of ‘new’ universal solutions.

## Introduction

A set of POR papers analysing COVID-19 responses, take as part of their explanatory tool kit how logics influences people and events. Christensen (2021) contrasts the logics flowing from March and Olsen’s (1983) homogeneous leadership, with those based on Selznick’s (1957) notion of homogeneous groups resulting in a situated logic of negotiation and compromise. Mattei and Vigevano’s (2021) analysis of the Italian COVID response interrelating national and local bodies, identifies a logic of underlaying policy integration cooperation that enhanced effectiveness. Mozumder (2021) suggests that the logic of learning from practice was made difficult in the UK because of diminished trust in agents and institutions.

Our paper picks up this issue of logics in how public organisations react to events. This is important as public managers increasingly focus on organising processes rather than organisational imperative: an essential change when most major policy responses now call for partnership, networks and/or ecosystem cooperation and integration. Situated and customised responses to events, taking advantage of available strengths and opportunities are perhaps particularly important, where user/client/customer feedback points to ways, as Normann (2002) notes, in which services can be improved. Our focus is on the potential conflict between the logics inherent in organisational form or organising in relation to the preferences of users and managers based on situated and social learning. Bourdieu (1984) and Bernstein (2000) emphasised how conduct of conduct rules (governance) are influenced by ‘soft’ socio-cultural practice, which they in turn reproduce and reshape. Faced with volatile and rapidly altering service environments, street-level decisions (Lipsky, 1980) can become patterned into ‘pop-up” governance-as-legitimacy (Laclau 1990).

Research about public organisations increasingly references logics: isomorphic logic (DiMaggio & Powell, 1983); public service logic (Ngoye et al., 2019; Osborne, 2017); management decision logics (Thornton et al., 2015) [ framing of public institutions and *rules of the game* logics (Scott, 2008); network management logics (Kooiman, 2003); and service-dominant logic (Lusch & Vargo, 2014, cf. Lopes & Alves, 2020). Bourdieu’s (1984) logic-of-practice is intended to ground logics in practice.

Logics is now a prominent idea in research on organisation from post-structural and socio-cultural process perspectives. March and Olsen (1989) for example, argued that *logic of appropriateness* has limited scope giving way to *logic of consequences*, apportioned by a hierarchy of logics. For social scientists seeking to generalise research results positing logics is an opportunity, provided as Kinder (2000) argues, they are recontextualised. Where logics are not re-grounded there is danger of assuming the future is dictated by yesterday, discounting human intervention. Determinism of this sort is a major issue in social research. Few social theorists now aim to ‘discover’ the ‘iron’ laws of society beloved by nineteenth century theorists. Logics properly applied are mediated by cognitive, emotional, (possibly) trusting people and especially so in services for people. As Jacobsen (2021) shows, in partnerships between the public and private sectors admixtures of potentially competing logics can be hybridised or combined to achieve public value.

Important thinkers criticise using logics from the perspective of diminishing agency include Wittgenstein (2001), Arendt (1951) and Chomsky (1969). Others highlight problems in agents choosing between multiple logics (Berman, 2012), or conflicting logics (Lounsbury, 2007), or over-reliance on logics to predict solutions (Dewey (1938), leading Archer (2000) and Toulmin (2003a, 2003b) for example to call for more research on the use of logics and contingency in social research.

Elder-Vass (2010) suggests a synthesis between Bourdieu’s (1990) idea of *habitus* as structuring thinking with Archer’s (2000) idea of *inner conversations* reflecting on choices in context. We find this synthesis inadequate for PM research since it unsatisfactorily addresses the complexity people face in public services and the nature of the learning processes they undertake, implying a clear view of how situated learning occurs and is used. Our research question is: are there negative effects of the use of logics in public organisation research on active human agency?

Our contribution is two-fold: we argue that Elder-Vass’ approach benefits from close synthesis with social learning theory (including recent thinking on trust, emotions, and distributed learning) and secondly, that grounding all usage of logics in logic-of-practice helps avoid a reification of logics. In pursuing these arguments, we build up a new conceptual model with which to approach logics in current research on organising in the public sector; suggesting ways in which frameworks using logics in can be strengthened.

The paper proceeds by exploring and defining the meaning of logics. Illustrating how the use of logics has become important in public organisation theory, one example being Bright’s (2021) recent use of Klijn et al.*,* (2016) *bureaucratic logic* to analyse how organisational identity interrelates with motivation. We argue that logics are only valid when grounded in situated experience from logic-of-practice in a particular service setting. Since contexts and cultures differ, it cannot be assumed that logics applicable in one setting are appropriate to another. We give a short exposition of how active agents in a particular setting learn logics and apply them using Vygotsky’s social learning theory. We then consider how logics are currently deployed in public organisation research taking the example of Vargo and Lusch’s (2008) service-dominant logic and Klijn et al., (2016) and Kooiman’s (2003) network management. We show that in both cases logics are regarded as universals; there is an absence of active human agency in both cases. From this we argue for a review of how logics and active agency and currently used in public organisation research citing logics.

## Logics: Genealogy and Use

Thornton and Ocasio (1999:804) define institutional logics as *the socially constructed, historical patterns of material practices, assumptions, values, beliefs, and rules by which individuals produce and reproduce their material subsistence, organize time and space, and provide meaning to their social reality*. Linking individual cognition to social structures they Thornton and Ocasio (2017) trace the idea to Selznick (1948) and later Zucker (1983), noting that Olson’s (1970) collective action emphasised individual consciousness and Fleck (1979) the notion of *thought style* as micro-social conditioning.

Douglas (1987:63) argued that organisations produce and reproduce *sameness* by embedding knowledge and when organisations interact, (DiMaggio & Powell, 1983) isomorphic logic diffused sameness, even according to Friedland and Alford (1991) across governances and between Governments. Wary of imitating symbolisms, Jackall (1988) argues imitating practice is more important; importantly suggesting the *embedded agency* is more important than imitative structures. Jackall’s framing of enablers and constraints continues to be cited though less is paid to the idea that only logics based on practice evidence have value.

Evidencing logics remains contentious for Toulmin (2003a:213) who says, *Warm hearts allied with cool heads seek a middle way between the extremes of abstract theory and personal impulse*, a wariness of deduced logics shared by Arendt (1958) who worries about *thoughtlessness* (1958:62) displacing empirical inquiry. Her emphasis on active agency (discussed below), including socially generated trust, is echoed by Popper (2007) who distrusts any logic not empirically founded.

Processes creating logics are subject to close scrutiny. For example, Van Benthem and Pacuit’s (2010) idea of *temporal logics* captures the point that logic in one time-frame may be non-logical in another. Epstein (1995) points to people internalising multiple logics and researchers need to justify their choices arguing that *splitting and splicing* of logics by agents are often unconscious. Kahneman and Tversky (1982) would agree, noting that slower (rational) selection of logics is post-facto justification of emotional preferences. Janik and Toulmin (1996) fear that transferring logics between locations is problematic.

### Learning and Logics

The idea of logics is widespread in public organisation research in justifying interpretations and actions as the recent POR articiles demonstrate. Dunn and Jones (2010) argue that following the introduction of new public management (NPM), Doctors adopt a new array of logics, including management heuristics and processes. Suggesting Finnish Doctors are more accepting than their Norwegian colleagues of NPM, Berg et al. (2017) suggest this due to identity change. Also investigating Doctors and NPM, Berg et al. (2017) argue that identity change is more profound amongst Norwegian than Finnish Doctors, since the former have less acceptance of NPM logics. It is now common-place for researchers to follow Scott (2008) and speak of logics in and between organisations or follow Freidson (2001) and comment on changing logics within professions without citing grounded empirical evidence. Bjerregaard (2011:195) argues that organisational logics are derived from institutional logics, giving a *hierarchical authority* of logics: these hierarchies of logics he says are somehow learned and accepted as justifying actions.

This short review supports our argument that new research into the use of logics in public organisation research is needed. Bourdieu (1990) and Zacka (2017) would support this conclusion; they draw attention to logic-of-practice – active learning by agents of patterned behaviour in contrast to the Habermasian deduction of logics from theory and their generalised usage. Bourdieu uses logic-of-practice to explain stability and change: practice-based habituations and frameworks and metaphors for thinking. Bourdieu’s logic-of-practice grounds logics in situated practice, not to be confused with Gidden’s (1984) use of the term for whom logic-of-practice results in new social structures.

We conclude that conceptual development in public organisation theory often features the idea of logics: isomorphic logic, public service logic, logics in management decision taking, logics in framing the *rules of the game*, logic in the management of networks, service-dominant logic. How conceptually robust, grounded and evidentially-situated are these logics in public organisation research? How do different logics relate to one another? We turn now to look further inside logics from the perspective of human agency.

## Agent-Centred Social Theory

Problematising logics in public organisation research and highlighting Bourdieu’s point that logics are learned, draws attention to active human agency as learners. Agency too is a contested idea: are cognition and intent essential characteristics, (b) what constitutes collective agency and (c) is agency contingent on context? Our perspective is that agency necessarily involves cognitive intent, and this precludes non-human ‘actants’ from agency, though in the special sense of distributed learning, collectives of people may be said metaphorically to possess agency.

Following Elder-Vass’s (2010) we agree that Archer’s (2003) *internal conversation* shaping agency and Bourdieu’s (1984) idea of *habitus* influencing agency are reconciled by the idea of *emergence* from complexity theory. This aligns with Morin’s (1959, 1982, 1986) contribution to active agency in French social theory, which can be underestimated; he spoke of *recursive causality*, in similar terms to Whitehead’s (1929) *being and becoming*: social order both creates and is created by active human agency.

Agentic acting with intent suggests intention-to-act (future) and intention-in-action (current activity). Intention therefore introduces psychological deliberation into agency, often as Elster (1979) notes from *pre-commitment* to particular goals citing ends-means coherence. Intention seeks control over future outcomes resulting from present behaviour; it is volition to act as Bratman (1987; 1991) says, based on cognitive reflection and/or beliefs, what Dewey calls *reflective intelligence*.

Since individuals are continually interpreting and responding to events and the activities of other agents, this catalyses new *flows of conduct* (Giddens, 1979:55) that continually emerge making human cognitive agency the micro-foundation of social research. Individual cognition and learning is then central to active agency, this the opposite of structural theory (Parsons, Althusser) which accords agency to non-cognitive structures such as bureaucracy and organisation. Similarly, we reject actor-network approaches (Latour, 1992), that attribute agency to non-human actants.

We employ Vygotsky’s (1986) social learning approach in which learning is social; featuring cognitions, relationality and emotions (especially trust); these mediate learning through the individual’s context and culture. Language, frameworks, concepts, social morès and norms influence learning. As Wertsch et al. (1995:25) says, *we can never speak from nowhere*. Learning cannot be reduced to bio-deterministic synaptic processing. Bernstein (2000) blends agent-centred and social learning approaches to explaining social change (see Hasan, 2001, 2005. He argues that restricted and elaborated codes of interpretation are the result of interaction between active agents’ identity and inherited cultural meanings. New social constructions, emergences that may constitute logics, can be weak or strong for Elder-Vass, yet always—as Arthur’s (2015) complexity theory suggests—arise from non-linear thinking and active processes with unforeseen results, the result of emotional attachments. Social learning aligns closely with Bourdieu’s logic-of-practice: concrete experiences and their interpretation by cognitive human agents, balance stability (morphostasis) and change (morphogenesis). Sense-making (which includes possible logics) are always provisional and transitory, since social life is always dynamically responding to the actions and ideas of other people and external events. Agentic intent then is ontologically founded on social learning, which is always relational and in arenas of complexity, continuous; context mediates without deterministically shaping learning and intent.

### Context and Active Agency

Cognitive human agents have both the capability to act and the capacity for cognition. Capability allows intent to result in social effects; those intended or not (Hvinden et al. 2018). Distinctive human agency (Ci 2011) is power, since intentions always subjectively *mobilises bias* (Schattschneider, 1975a, 1975b). All agents operate in domains in which this subjective power is recognised as plausible (or not); contexts therefore give content (intention) and modality to agency. As Kiser (1999) argues this makes agents and their context inseparable. “Logic” becomes an accumulation of behaviour at an individual level from which the individual learns; it is not a superstructural imposition.

Agency is relational, enlivened only in social processes (Burkitt, 2016). It is in Whitehead’s (1929) terms *becoming*, not *being*: an emergence that is coproduced (Weber, 2006). The interactivity of agents creates intent not something inherent in objects (Dépelteau 2010). This perspective closely aligns with relational sociology, which focuses on processual relationships (Emirbayer and Mishe 1998; Daniels, 2001). Unlike Vandenberghe (2010), who suggests human agents can operate without prior intent, we follow Gergen (2009 and Archer (2012; 2013) who insist that agency presumes reflexivity, which by iterational *morphogenesis* explains how agents both produce *and* reproduce social structures. Social structures are important Elder-Vass (2010) argues not because they dictate logics, but rather because they mediate learning, which may become logics: social structures cannot act independently of human intent.

Elder-Vass (2010, 2007a, 2007b) points out that although at first sight (a) Archer’s (2003) emphasis on reflexivity (*internal conversation*) shaping agency (and creating personal and social identity), and (b) Bourdieu’s (1984) accent on habitus as socially conditioning (generative capacity to produce, reproduce, change), appear irreconcilable, the idea of emergence (from complexity theory) reconciles the two approaches. Agency itself is always emergent, always *becoming*. Welcoming these ideas, our view is that further steps are needed to explore how the social learning processes occur the give rise to and interpret emergences, leading us towards Vygotskian social learning.

### Collective Agency?

What then of collective agency, such as Marx’s (1852) *class for itself* or collective unlearning in public organisations (Stenvall et al., 2018), or corporate responsibility? Law often ascribes moral responsibility to corporate bodies. However, the agency of collective bodies (state, working-class, companies) is quite different from individual agency, which presumes cognitive ability and as Arendt (1969) argued, collective actions cannot abrogate the inalienable culpability for their actions. Nor can agency be confused or conflated with finance principal-agency theory, which often ignores context and presumes rational choice (Becker, Williamson). For Perrow (1990:121) this approach is *not only wrong but also dangerous*. Since agency presumes learners and intent, collective agency such as learning organisations are illusory – organisations cannot learn, since only individual cognisant individuals can think.

Grounded logics are the human/social construction working on nature and in social relationships, the dialectical logics Marx developed in Capital (1993), applied historically (1852) and justified philosophically (1976), grounded in his theory of capitalism (1973) and which Engels (1859) related to dialectics of nature. Whereas some social theorists (Schatzki 2019 being an example) *sayings and doings* shape social change, Marx (1973) that it is not ideals (ideas ungrounded in material practice) that drive social change. Instead, humans being the only animal capable of advanced cognition, including intentionality (design) and purposive labour enhancing the productivity of nature, we use self-consciousness to create social consciousness. Shared with others and becoming collective intentionality and consciousness, social change results. Humans are capable of thought-through collective agency. It is from patterns of agency that new logics are created and in turn, individual cognitions and collective intent make use of previously formulated logics. Logics then are actively constructed, quite unlike abstracted ideas such as Kant’s imperative or Smith invisible hand.

Whereas individual consciousness is the result of cognition and affect (Vygotsky, 1986), collective consciousness is embedded from external sources. Blackmore’s (2003) memes and Durkheim’s (1893) analysis of religious show this occurring in wider society. In organisations and organising however, Wittgenstein’s *follow the rules* is replaced by *follow the leader*, since as Schattschneider (1975a, 1975b:71) says, organisations are the *mobilisation of bias* they necessarily privilege certain outcomes. Collectivities of people in organisations instead of following an ideology, and ideal in Ilyenkov’s (2008) terms, require a dynamic narrative connecting problem and solution, creating organic solidarity: collective consciousness in organisations is necessarily highly situated and, where leadership is effective, is guided towards understanding the germ-cell or essence of the problem, recognising contradictions in the current state-of-affairs, and takes collective active to achieve what the leader has explained as a preferred solution (Prilleltensky (1997:525). Individuals still must make sense of the leader’s narrative; often this is helped by framing, frameworks, metaphors and language supplied by the leader. Part of the leader’s role in ecosystems is legitimising the other agents in the ecosystem, with whom the organisation’s members must work in order to deliver the preferred solution. For Blunden (2015) effective project work in organisations relies on leadership building this collective consciousness. Such leaders will combine vision with the leadership, management and administrative competences to which Hartley and Allison (2000) refer. Creating collective consciousness in a public or other organisation, requires a leader able to make sense of problem–solution in context and culture and to marshal the collective into activity.

One of the more contentious results of what Bernstein (2000) terms the *cultural turn* in social theory, is the attribution of agency to culture. As Harvey (1982) and others make clear, scaling between levels of analysis only makes sense if not employed as a deterministic hierarchy of simplified causalities from the general to the particular.

### Socio-Cultural Theory

**Fig. 1:**
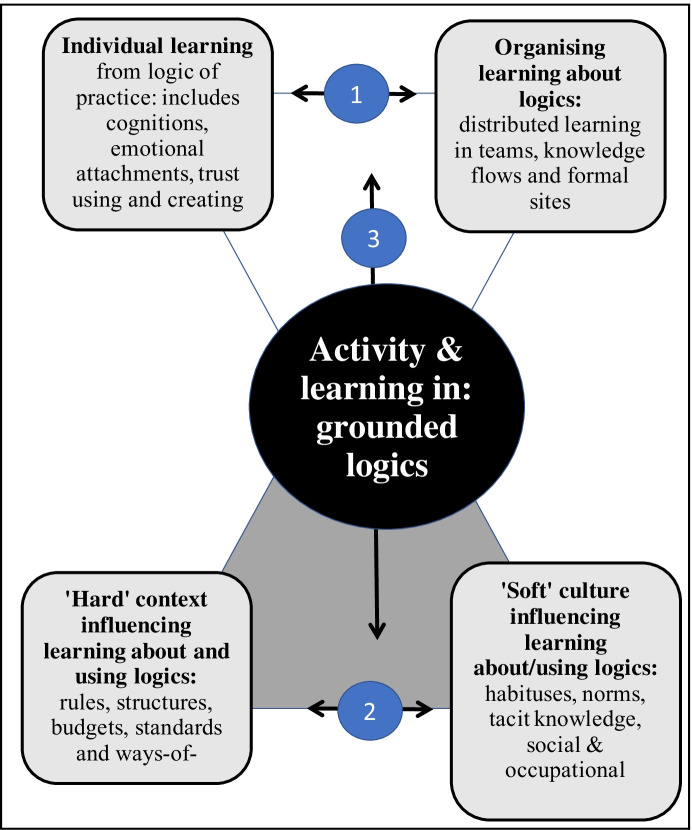
Simplified Vygotskian social learning framework (derived from Illeris, 2004; Engeström (1996, 1996) and Nardi (1996)

**Fig. 2:**
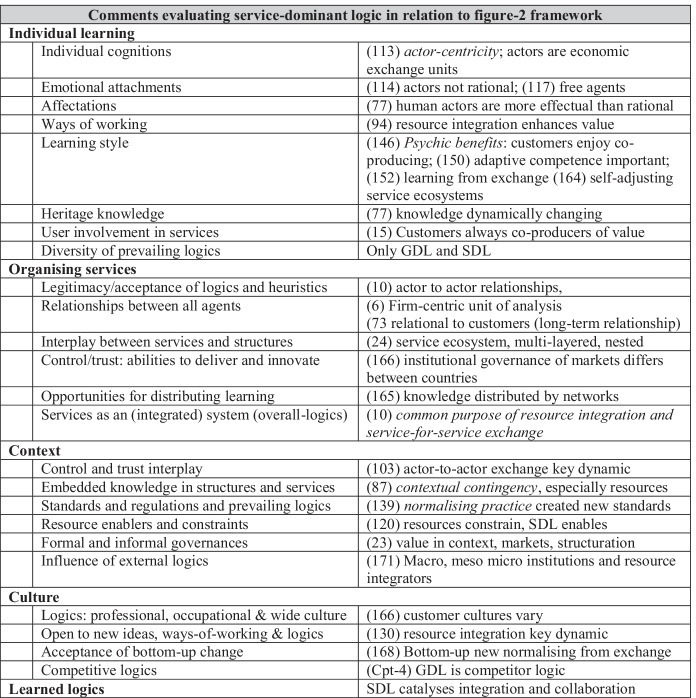
Simplified Vygotskian social learning framework (derived from Illeris, 2004; Engeström (1996, 1996) and Nardi (1996)

Learning and deploying new knowledge is then an essential aspect of active agency: learning patterns of activity from logic-of-practice creates new bottom-up governances and learning from user feedback helps personalise the design and delivery of local public services. How then does this occur? Figure 1 is a simplified exposition of how social learning occurs using Vygotsky’s (1986; 1997) perspective and drawing on the work of Engeström et al., (1999), Nardi (1996) and Illeris (2004). Individual thinking is shaped by logics and in distribution new logics are formulated, in turn influenced by context and culture. Arrows 1, 2 and 3 indicate these interactions constituting the activity centre in the middle of Fig. 1 illustrating how logics influences learning and in turn by learning and patterned practice, new logics are created. A key point is the sense-making by cognitive individuals references their emotional attachments both to old ways-of-working and to a vision of how new services and governances might operate drawing on heuristics (thinking frameworks) evolved from formal education and in practice. As Vygotsky (1986:282) says, *[Thought] is not born of other thoughts. Thought has its origins in the motivating sphere of consciousness, a sphere that includes our inclinations and needs, our interests and impulses, and our affect and emotions* Figs. 2 3.

**Fig. 3:**
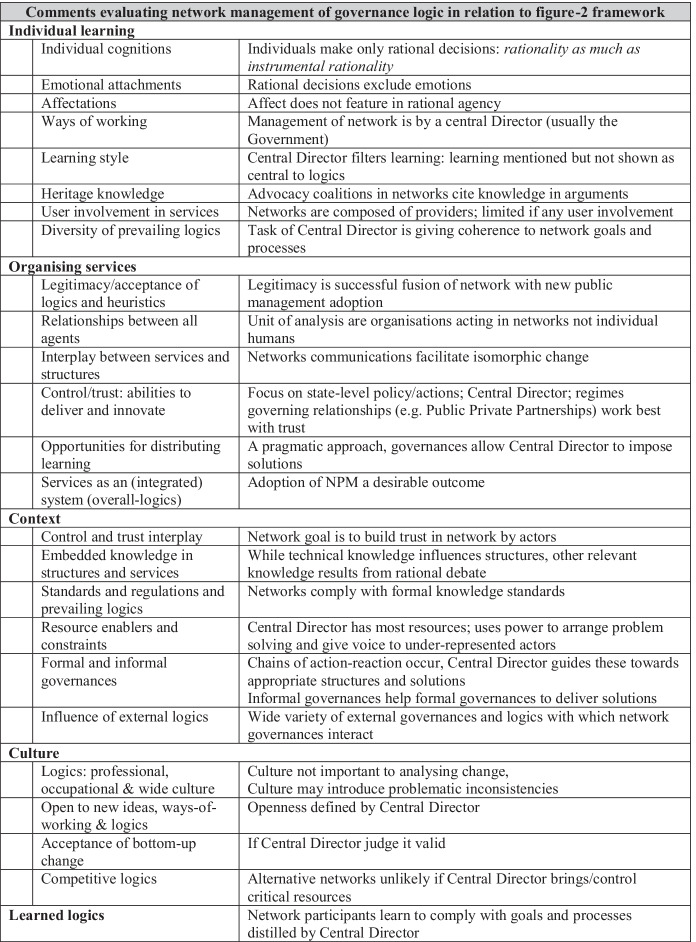
Simplified Vygotskian social learning framework (derived from Illeris, 2004; Engeström (1996, 1996) and Nardi (1996)

Individual learning (top-left) is distributed during organising service delivery (top-right). All learning occurs in a specific context (bottom-left) meaning ‘hard’ rules and norms such as budgets, regulations, ethical standards and (bottom-right) ‘soft’ cultural norms such as general social culture, occupational culture. While taking account of ‘objective’ facts, the individual learning is non-rational.

Trust is an especially important emotion in individual learning and its distribution for PM since the services target vulnerable people reliant on trust and trust accompanies representatives between sets of professionals. As Weibel and Six (2013) argue, this willing acceptance of vulnerability between agents in turn presupposes relatedness between agents, competence and autonomy. Trust and control are in once sense opposites and in another complementary. More trust in a service system means less need for formal management structures and oversight accountability. When trouble occurs Six (2005) notes, trust is either dissipated or strengthened.

Social learning then is relational and offers a way of improving Elder-Vass’s position by explaining how agent learning reframes and reformulates logics in situated theorisations of public service dynamics.

## Logics in Public Service Organisation Research

Taking two oft-cited uses of logics in public organisation research as examples, here we consider how far they are grounded in learning by active agents.

We have chosen (a) Vargo and Lusch’s (2004, 2007, 2008 and 2017) service-dominant logic and (b) Kooiman (2003); and Klijn and Koppenjan’s (2014) management of networks. We choose Vargo and Lusch and Kooiman and Klijn because they will be familiar to readers, and each apply logics at the level of organisations delivering services. The choices are for illustrative purposes; we are not in any way suggesting that these pieces of research are other than valuable. In each case, we present a table of how logics are referenced in these two bodies of research relative to social learning by active agent in logic-of-practice.

### Service-Dominant Logic and Logic Evaluation

Vargo and Lusch (2004, 2007, 2008, 2017) offer an integrated marketing perspective on services, arguing that value (in use) is co-created by service providers and users; focusing on private services little mention is made of public services. They argue that a goods-dominant logic (GDL, featuring tangible goods, a supply-side mindset and objective success criteria) is being superseded by a SDL, which concentrates on intangible services, a customer-focused mindset and subjective success criteria: goods are a distribution mechanism for services. Our focus here is on the use Vargo and Lusch make of logics and the extent to which the logics are grounded in practice and agent learning.

Using Lusch and Vargo (2014), a 224-page exposition of SDL, Table 1 summarises the stance taken in relations to factors constituting grounded logics. In this welcome exhortation to adopt customer-focused activity, non-exchange (public and some 3S) people and public organisations are seldom mentioned. New service dominant logics arise in abstract exchange relationships. Few individual service providers or people are mentioned; the logics are derived from market exchange. SDL is presented as a new paradigm evolving from firms’ exchange activity, without any reference to the people constituting the firms. For example, new knowledge arises from exchange, without mention of learning, cognition, affectations. The SDL world is populated by firms exchanging services with other firms and customers, none of which reference in any detailed way the context and culture in which the exchanges occur. SDL is a switch from GDL the processes of which do not feature human agentic involvement.

**Table 1:** Logics in service-dominant logic (page references from Lusch and Vargo 2014)

Comments evaluating service-dominant logic in relation to Table-1 framework		
Individual learning		
	Individual cognitions	(113) *actor-centricity*; actors are economic exchange units
	Emotional attachments	(114) actors not rational; (117) free agents
	Affectations	(77) human actors are more effectual than rational
	Ways of working	(94) resource integration enhances value
	Learning style	(146) *Psychic benefits*: customers enjoy co-producing; (150) adaptive competence important; (152) learning from exchange (164) self-adjusting service ecosystems
	Heritage knowledge	(77) knowledge dynamically changing
	User involvement in services	(15) Customers always co-producers of value
	Diversity of prevailing logics	Only GDL and SDL
Organising services		
	Legitimacy/acceptance of logics and heuristics	(10) actor to actor relationships,
	Relationships between all agents	(6) Firm-centric unit of analysis
(73 relational to customers (long-term relationship)		
	Interplay between services and structures	(24) service ecosystem, multi-layered, nested
	Control/trust: abilities to deliver and innovate	(166) institutional governance of markets differs between countries
	Opportunities for distributing learning	(165) knowledge distributed by networks
	Services as an (integrated) system (overall-logics)	(10) *common purpose of resource integration and service-for-service exchange*
Context		
	Control and trust interplay	(103) actor-to-actor exchange key dynamic
	Embedded knowledge in structures and services	(87) *contextual contingency*, especially resources
	Standards and regulations and prevailing logics	(139) *normalising practice* created new standards
	Resource enablers and constraints	(120) resources constrain, SDL enables
	Formal and informal governances	(23) value in context, markets, structuration
	Influence of external logics	(171) Macro, meso micro institutions and resource integrators
Culture		
	Logics: professional, occupational & wide culture	(166) customer cultures vary
	Open to new ideas, ways-of-working & logics	(130) resource integration key dynamic
	Acceptance of bottom-up change	(168) Bottom-up new normalising from exchange
	Competitive logics	(Cpt-4) GDL is competitor logic
Learned logics	SDL catalyses integration and collaboration	

Our point is this: while Lusch and Vargo refer often to being actor-centric, there are few people in their exposition of logics and almost no public sector. The logics arise from market exchange between economic units (firms) within ecosystems and institutions envisaged from a market exchange perspective. There is no human thinking, feeling, or (human) relations agency. Agency is ascribed to inanimate entities: abstracted customers and firms. Although the services ecosystems self-adjust, they appear to do so without human decisions, responses, creativities. The only practice referenced are those of resource integration and market exchange; not real people, using real services. No competitive or conflicting logics exist, apart from GDL. This new SDL paradigm exists without reference to particular and situated contexts and cultures. SDL is a metaphysic, a belief system not grounded in practice or reality, not subject to verification or disproof. The logic is without reference to human intervention and can only be categorised as deterministic.

### Network of Management Governances and Logic Evaluation

Kooiman’s 249-page exposition (2003) and later work including Klijn (2008) and Klijn and Koppenjan (2014) presents a logic for governance analysis based on management of networks, used in a 427-page study of fisheries governance (2005). Kooiman and Bavink, (2013) sets out to explain how interdependency and relationships between agencies can best operate as society becomes more complex, dynamic and diverse. Table 2 summarises the stance taken by network management theorists towards grounding logics in practice and learning.

**Table 2:** Logics network management of governances taken from Kooiman (2003); Klijn (2008) and Klijn and Koppenjan (2014)

Comments evaluating network management of governance logic in relation to Table-1 framework		
Individual learning		
	Individual cognitions	Individuals make only rational decisions: *rationality as much as instrumental rationality*
	Emotional attachments	Rational decisions exclude emotions
	Affectations	Affect does not feature in rational agency
	Ways of working	Management of network is by a central Director (usually the Government)
	Learning style	Central Director filters learning: learning mentioned but not shown as central to logics
	Heritage knowledge	Advocacy coalitions in networks cite knowledge in arguments
	User involvement in services	Networks are composed of providers; limited if any user involvement
	Diversity of prevailing logics	Task of Central Director is giving coherence to network goals and processes
Organising services		
	Legitimacy/acceptance of logics and heuristics	Legitimacy is successful fusion of network with new public management adoption
	Relationships between all agents	Unit of analysis are organisations acting in networks not individual humans
	Interplay between services and structures	Networks communications facilitate isomorphic change
	Control/trust: abilities to deliver and innovate	Focus on state-level policy/actions; Central Director; regimes governing relationships (e.g. Public Private Partnerships) work best with trust
	Opportunities for distributing learning	A pragmatic approach, governances allow Central Director to impose solutions
	Services as an (integrated) system (overall-logics)	Adoption of NPM a desirable outcome
Context		
	Control and trust interplay	Network goal is to build trust in network by actors
	Embedded knowledge in structures and services	While technical knowledge influences structures, other relevant knowledge results from rational debate
	Standards and regulations and prevailing logics	Networks comply with formal knowledge standards
	Resource enablers and constraints	Central Director has most resources; uses power to arrange problem solving and give voice to under-represented actors
	Formal and informal governances	Chains of action-reaction occur, Central Director guides these towards appropriate structures and solutions
Informal governances help formal governances to deliver solutions		
	Influence of external logics	Wide variety of external governances and logics with which network governances interact
Culture		
	Logics: professional, occupational & wide culture	Culture not important to analysing change,
Culture may introduce problematic inconsistencies		
	Open to new ideas, ways-of-working & logics	Openness defined by Central Director
	Acceptance of bottom-up change	If Central Director judge it valid
	Competitive logics	Alternative networks unlikely if Central Director brings/control critical resources
Learned logics	Network participants learn to comply with goals and processes distilled by Central Director	

For Kooiman networks are the preferred structure to solve problems and a Central-Controller led governance manages the network. Central Controllers, usually Government, bring resources to problem-solving; networks are characterised by rational-cognitive agency and reference formal knowledge in decision taking. NPM efficiency is a desirable goal of the networks. Networks form governance hierarchies with first-order governance (day-to-day), institutional arrangements (second order) and (third order) meta-governance (similar to Habermas’ communicative rationality. Network governance is appropriate to policy networking, inter-organisational delivery of service and policy implementation: it is not confined to second-order policy making. The logic in network management then is compliance with the preferences of the Central Controller; often the targets and processes associated with new public management.

To assess the implications of Table 2 in steps. Kooiman and his colleagues argue that governances are best analysed and constructed as networks, that networks are best centrally directed, network participants ought to act and think rationally, the possessing resources (power) gives legitimacy to network central directors and that these logics apply whatever the context and culture.

Network management as a logic is not grounded in active agency; it is not derived from lessons learned by cognitive-emotional humans. The approach appears to be a quirky ideal type justifying the adopting of new public management and legitimating the power of central Government in public service design and delivery.

## Discussion and Conclusions

Tables 1 and 2 illustrate little evidence of learning by active agents and little evidence of grounding their prescribed logics in practice. Why is this absence of social learning in SDL and the Rotterdam group’s network management approach important? The answer is that social research bereft of cognisant people is questionable: the social constructions resulting from such research are from inside the mind of the researchers not logic-of-practice.

It is of course possible to argue that logics are an implicit or sub-conscious aspect of agent behaviour. None of the theorists mentioned take this position. Assuming an unverifiable set of beliefs would open new methodological cans of worms. Does this invalidate the approaches suggested by SDL and network management? Not necessarily, however, it makes it important to know the epistemic stance of the researchers.

Both Lusch and Vargo and Kooiman and his colleagues are suggesting that there are alternative mindsets to (for example) market transactionality (price) or hierarchy (power). Both sets of researchers are proposing new principles to guide thinking and action. Our central point is that each offer a play without actors, a world without people; principles decided deductively not grounded, ways of operating where the key units of analysis are not cognitive-emotional persons, but instead firms/exchanges (SDL) or organisations in networks.

Both SDL and network management are logics oft cited in public organisation research. Llewelyn (2003) argued that there are five types of theorising available to qualitative researchers: (a) metaphors; (b) differentiation; (c) conceptualisation; (d) context-bound theorising of settings and (e) context-free ‘grand’ theorizing. Both SDL and network management are context-free theorising: meta-narratives, in each case produced from deep conceptual reflection on enduring social relationships and causalities of how structures and agency interact. The fact that such theorisations are not derived from empirically substantiated agent learning that grounds logics in practice, does not in itself deprive them of usefulness.

Nonetheless, deduced theorisation should be acknowledged, and the assumptions laid bare; so that when applied to particular types of human agency or situated contexts and cultures, it is clear what evidence from structures and agency are appropriate for researchers using these approaches to seek. If there are contexts and cultures to which SDL and network management do not apply, then this too should be acknowledged to avoid using the approaches to inappropriately frame research problems and/or embed assumptions in empirical work unknowingly. This is especially important for public organisation research which is international in nature, transgressive of contexts and cultures and needs to know if conceptual tools are proposed as universally applicable or if of limited generalisability, the nature of the limitations. Our own view is that the logics in SDL is a metaphysic and network management epistemologically flawed, given its assumptions of rationality and privileging of power. Neither approach is universally applicable—contexts and cultures vary considerably.

General theories are stronger on explanation (attributed causality, i.e. why) and weaker on understanding (i.e. what—identifying the existence of outcomes predicted by the theory); since social research phenomena are constructions. This can lead to confusing the map with the terrain i.e. finding what one sets out looking for, making theory falsification impossible. This of course is the great advantage of grounding social research by investigating agent interpretations and actions: instead of beginning with a theory and seeking evidence of its usefulness. As Glaser and Strauss (1967:2) propose grounded theory discovers theory from systematic data. Groundedness itself being a metaphor for linking to a feedback loop, such as ethnographic studies or qualitative interviews producing case studies. For Archer (2000) grasping both agency and structures is essential to explaining social activity: neither SDL nor network management do this.

We conclude that some researchers continue to find Lusch and Vargo’s SDL and Kooiman’s network management logics, or theories, useful. Their use however should be constrained by a clear understanding of how the key concepts relate to the particular context and culture being studied. Further, researchers should note that in focus on logics comes at the cost of not focusing on human agency – a controversial choice in public management research. As Fig. 1 illustrates use of logics is not passive, from using logics new logics emerge addressing contradictions and conflicts in the previous patterns of logics, as they apply to current problems.

For public organisation managers the clear implication of this research is to avoid off-the-shelf, transformative, paradigm-switching new tools. There is no alternative but deeply investigating problems in situation, digging to identify the germ-cell essence of any problem and to propose solutions accordingly. Public manager should always to wary of universal tools or solutions and instead be unafraid to conclude that their organisation, their problem requires tools of analysis and solutions uniquely suiting their organisation and its capabilities. Our contribution revolves around viewing logics not as passive ‘ideas’ isolated from practice, but instead as ‘actively’ helping to socially construct what current practice is and thereby create new logics. In short, logics occupy a dialectical place in learning and problem solving and are not static nor fixed.
